# Timing somatic events in the evolution of cancer

**DOI:** 10.1186/s13059-018-1476-3

**Published:** 2018-07-24

**Authors:** Clemency Jolly, Peter Van Loo

**Affiliations:** 1 The Francis Crick Institute, 1 Midland Road, London, NW1 1AT UK; 2 Department of Human Genetics, University of Leuven, B-3000 Leuven, Belgium

## Abstract

Cancer arises through the accumulation of somatic mutations over time. An understanding of the sequence of events during this process should allow both earlier diagnosis and better prediction of cancer progression. However, the pathways of tumor evolution have not yet been comprehensively characterized. With the advent of whole genome sequencing, it is now possible to infer the evolutionary history of single tumors from the snapshot of their genome taken at diagnosis, giving new insights into the biology of tumorigenesis.

## Introduction: the evolution of cancer as a multistage process over time

The risk of developing cancer increases steadily throughout an individual’s lifetime, rising steeply from middle age onwards. In the 1950s, Armitage and Doll [1] proposed that the observed trends in cancer incidence would be consistent with the progression of carcinogenesis through a series of six or seven sequential cellular aberrations. The cumulative effect of mutations during cancer development then went on to be explicitly demonstrated in seminal work on retinoblastoma, in which two mutational events are required to initiate tumor formation, inspiring Knudson’s “two-hit” hypothesis [2]. By the late 1970s, an overall picture of cancer development was beginning to emerge, formalized in the clonal model of tumor evolution proposed by Nowell [3], which is still widely accepted today. Essentially, the evolution of cancer may be considered as a Darwinian process; mutations randomly accumulate in the genomes of normal cells and, where advantageous, result in clonal expansions as a product of natural selection [4].

In the last few decades, key genes have been identified that are frequently aberrated in the cancer genome, either through traditional molecular genetics approaches or more recently from next-generation sequencing [5–7]. Little is known, however, of the timing of somatic mutations, or the order in which they occur during tumor evolution. In 1990, Fearon and Vogelstein [8] were the first to address this question in a landmark study of colorectal tumors, charting the acquisition of point mutations and copy number changes during the progression from normal epithelial tissue to carcinoma and metastatic disease. Since this work, others have attempted to reconstruct similar pathways of tumor evolution for other tissue types using the same approach, typically by comparing the genomic aberrations present in different tumor samples, either between precursor lesions and the resulting tumors, or across cohorts of patients with different stages of disease [9–12].

In recent years, such cancer progression analyses have been further advanced by the application of mathematical models such as oncogenetic trees and directed acyclical graphs [13]. Cancer genome sequencing, however, now allows a much more direct study of tumor evolution within single patients from temporally or spatially separated samples [14–16]. Furthermore, the development of algorithms for reconstructing a tumor’s evolutionary history has made it possible to infer the timing of specific mutations, and to characterize a sequence of events, from whole genome sequencing of single biopsies [17].

Deciphering the temporal sequence of events as cancers develop and progress is essential for a comprehensive understanding of tumorigenesis, and for identifying the earliest events in tumor evolution. This may provide markers for faster diagnosis and treatment, as well as improving our ability to predict cancer progression. Here, we review the various approaches for examining tumor evolution, including current methodologies for timing mutations, and describe how this has advanced our understanding of tumor biology.

## Timing events across multiple tumor samples

Taking multiple samples of a tumor, separated either in space or time, provides the most direct approach for studying tumor evolution. By comparing the cancer genome at different stages of development, one can immediately observe a sequence of events as the cancer progresses. Much information can already be gained from each sample, as sequencing data, both whole genome and exome, contain a detailed catalogue of the somatic mutations that the cancer genome has acquired. Furthermore, the underlying clonal architecture of the bulk tumor sample can be inferred from the variant allele frequency (VAF) of somatic point mutations [17, 18], which itself gives insight into the earlier and later stages of evolution. Clonal mutations common to all sampled tumor cells must precede subclonal mutations, which are only present in a fraction of the sampled tumor cells. Thus, differences in the mutational profiles, or changes in the clonal composition of separate tumor samples, reveal how the cancer is developing over time [19, 20].

It can be informative to examine differences in the cancer genome at key stages during tumor progression, for example by comparing primary tumors with metastases, samples taken upon relapse, or with their precursor lesions. Many studies have compared the genomes of primary tumors with their corresponding metastases, often observing substantial evolutionary change accompanying the spread of disease, with metastases accumulating new mutations in addition to those they have carried forward from the original tumor [21–29]. These metastasis-specific mutations give insight into the final phases of tumor evolution, as the tumor cells move from the primary site and begin to develop in a new tissue.

In breast cancer, for example, the enrichment of *JAK2* and *STAT3* mutations in distant metastases relative to the primary tumor suggests their involvement in facilitating tumor progression and dissemination [28]. Specifically, these genes encode components of the JAK-STAT signaling pathway, which has been suggested to allow advanced metastatic tumors to evade the immune response [28]. In a recent large-scale study of clear-cell renal carcinoma [29], chromosomal aberrations were shown to play an important role in the process of metastasis; specifically, losses of 9p and 14q, which potentially drive metastasis through the interaction between *CDKN2A* (located on 9p) and *HIF1A* (14q). Similar studies of metastases from prostate [24, 25], skin [27], and pancreatic tumors [26], as well as metastases to the brain from various organs [23], have identified an enrichment of alterations to *TP53* and androgen receptor genes, β-catenin (*CTNNB1*), *CCNE1*, and *MYC*, and perturbation of the PI3K/AKTmTOR and HER2/EGFR signaling pathways, respectively.

Samples taken from primary and recurrent tumors give similar insight into the genomic changes accompanying tumor progression, either in response to, or in the absence of, treatment [30–33]. Where tumors have been treated with chemotherapy, this allows the characterization of events that have occurred in the course of subsequent tumor evolution, and which may have conferred therapeutic resistance. For instance, Patch et al. [34] observed relapse-specific lesions, including *BRCA* reversions, methylation changes, and promoter fusion events, contributing to a platinum-resistant phenotype across a cohort of ovarian cancers. Furthermore, taking multiple samples as a time series throughout the course of a patient’s disease progression allows a particularly fine-grained study of tumor evolution. Applying this rationale, Schuh et al. [14] took five time points each from three patients with chronic lymphocytic leukemia, typically before or after new courses of treatment. This allowed them to precisely track clonal evolution over time, monitoring the dynamics of subclonal cellular populations in response to treatment, and identifying putative founder events based on their frequency across the time series.

Looking towards the initial stages of tumorigenesis, comparisons between primary tumors and their corresponding precursor lesions [35–40], or even normal tissue [41], give insight into the very earliest cellular aberrations. This is an informative transition point in the evolution of a tumor, as the events common to both precursor and primary lesions may be examined for cancer progression risk markers, while those seen only in the tumor samples may represent events that are transformative to normal cells. Where it is possible to identify mutations in the normal tissue of healthy patients, corresponding to the tissue type of a given tumor, further distinction may be made between specific driver mutations, and the random passenger events that accumulate as a function of age [38].

Precursor lesions with paired primary tumors have been studied across various tissue types, including germ-cell [35], endometrial [36], and skin tumors [37], as well as hematological malignancies [30]. One of the best characterized examples of precursor lesions leading to tumor formation is Barrett’s esophagus, a condition caused by chronic acid reflux, conferring an increased risk of esophageal adenocarcinoma [39, 40]. Studies of Barrett’s esophagus compared with esophageal adenocarcinoma show that the process of neoplastic transformation is highly heterogeneous. Large numbers of mutations have been observed in precursor lesions and the resulting tumor, both largely dominated by C > A transversions, of which varying fractions are shared [39]. From precursor lesions, mutations in tumor suppressors such as *TP53* have been identified as early events, as they are common across biopsied regions of the esophagus and present prior to whole genome duplication. On the other hand, oncogenic activating mutations occur later, suggesting that these are amongst the subsequent steps required for the transformation of Barrett’s esophagus to an invasive adenocarcinoma [40].

As it may not always be possible to obtain repeated samples from a single patient, examining multiple regions from a single biopsy can give an alternative insight into tumor evolution, as one can infer a temporal sequence of events from their spatial distribution across the tumor. Furthermore, the phylogenetic trees of cancer evolution reconstructed from multiple regions of a single sample can incorporate much more detail than those from a single biopsy, which may miss subpopulations only present in certain regions of the tumor. Similar to serially acquired samples, multiregion sequencing allows the comparison of shared and private mutations between tumor regions to determine the ordering of events in cancer development [15, 16, 42–44]. For example, across 100 non-small-cell lung cancers, Jamal-Hanjani et al. [15] observed early clonal driver mutations in canonical driver genes such as *EGFR*, *MET*, and *BRAF* in adenocarcinoma, *NOTCH1* in squamous cell carcinoma, and mutations in *TP53* common to both cancer types. Late clonal and subclonal mutations, on the other hand, tended to have a wider variability, encompassing many genes involved with chromatin remodeling and DNA repair pathways.

Studies of multiple tumor samples can, therefore, give a detailed picture of the tumor genome and the changing dynamics of clonal populations. They are, however, typically limited to small sample sizes, particularly for solid tumors that are difficult to sample repeatedly, which means that it is not possible to extract general trajectories of evolution for a cancer type as a whole. Additionally, it is often financially impractical to apply whole genome sequencing to many samples across a number of patients in a cohort, and a compromise must often be sought by targeted or whole exome sequencing, which does not provide a complete picture of the tumor genome.

Single cell sequencing also represents a powerful approach for resolving intratumor heterogeneity and for investigating the later stages of tumor evolution [45–47]. Bulk tumor sequencing data do not have sufficient resolution for the detection of very low allele frequency mutations, and so one cannot characterize the very outermost branches of the phylogenetic tree. Single cell sequencing technologies, often in parallel with bulk sequencing experiments, now permit genotyping or calling of point mutations, large copy number aberrations, and structural variants in individual cells [48–51], which allows the creation of previously unattainable, highly detailed phylogenetic trees [52]. Although still at relatively early stages, such techniques are already providing key insight into the modes of tumor evolution. Single nucleus sequencing of triple-negative breast cancers, for example, has shown clonal dynamics consistent with early catastrophic copy number alterations, followed by long periods of evolutionary stasis, which would indicate a punctuated rather than gradual process of evolution in this tumor type [53].

## Reconstructing a tumor’s evolutionary past from a single sample

**Fig. 1:**
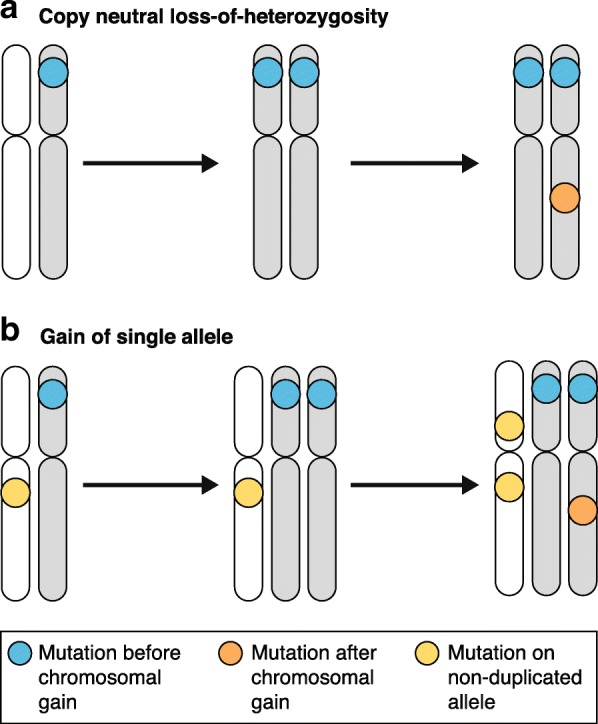
Timing copy number gains using point mutations. The relationship between point mutations and surrounding copy number gains can be used to infer the time of the gain. **a** Timing copy-neutral loss of heterozygosity (CNLOH). Blue mutations occurred prior to the CNLOH event, orange mutations occurred afterwards on either chromosome. **b** Gains of a single allele, where the other is retained, are more complex because single copy mutations can reflect both mutations that have occurred since the gain, and those on the non-duplicated allele (yellow)

Even when only one tumor sample may be obtained, there can still be plenty of information in the sequencing data to allow the inference of an order of events during tumor development. As discussed above, point mutations may be classified as clonal or subclonal based on the fraction of cancer cells that bear the mutation, and it is inferred that clonal mutations precede those that are subclonal. Furthermore, clonal mutations within regions of clonal chromosomal gains may be temporally divided into those that have occurred before the gain, and those that have occurred after [54, 55]. This relationship between point mutations and the surrounding copy number can be inferred from the variant allele frequency of the mutation, after taking into account the tumor purity and copy number to obtain the number of chromosomes carrying the mutation [18]. Mutations on two alleles must already have been present and were duplicated with the surrounding region (termed “early”), whereas those on a single allele must either have happened afterwards (termed “late”) or occurred on a non-duplicated allele (see Fig. 1).

Thus, it is possible to estimate the relative timing of individual mutations during the clonal phase of tumor evolution. Timed mutations within clonal copy number gains can then be used to estimate when the gain itself occurred, calculated from the rates of early and late mutations (see Box 1) [54]. If there are very many mutations carried by two alleles, and few carried by a single allele, this suggests that point mutations accumulated in this region over a longer period of time prior to the gain, which happened towards the end of clonal evolution. Conversely, if the majority of mutations are only carried by a single allele, this indicates that, proportionally, the gain occurred earlier in clonal tumor evolution. It is important to note that this approach does not assume a constant mutation rate, and thus time estimates correspond to “mutational time”, i.e., the timing of the gain indicates its relative position compared with point mutations.

In recent years, algorithms have been developed to implement this concept, using either a partial or full maximum-likelihood approach to first estimate the copy number of individual mutations, and then to use them to estimate the timing of chromosomal gains [56, 57]. As the number of chromosomes in existence at each stage of clonal tumor evolution must be accounted for when determining the mutation rate (see Box 1), it is important to be able to model the exact sequence of events during the acquisition of the chromosomal gain, and therefore simple gains lend themselves more easily to this approach. As suggested by Purdom et al. [56], these include regions of copy-neutral loss of heterozygosity (CNLOH), gains of a single allele, and double gains of a single allele. Whole genome duplication (WGD) events may be considered as an exceptional case, as one assumes that both alleles are gained simultaneously, although inevitably, on occasion, this will not hold true. As a caveat, it should be noted that only gains may be studied using this method; the mutations required to time chromosomal losses are lost with the chromosome itself.

Applying this approach to cancer genomes therefore gives insight into the timing of chromosomal gains, and the relative ordering of mutations, during the clonal evolution of a tumor, potentially highlighting the different mechanisms underpinning tumor development. For example, Nik-Zainal et al. [17] used this approach to time chromosomal gains during the evolution of 21 breast cancers. This study demonstrated that copy number gains are unlikely to be the first event during the evolution of breast cancer, but accumulate over time, with most gains occurring after the first 15–20% of mutational time. The quantitative time estimates of clonal duplications could then be integrated with the corresponding relative timing of other events, such as amplifications of *ERBB2*, *MYC*, and *CCND1*, and punctuated events such as chromothripsis, recapitulating the sequence of events throughout clonal evolution for this cohort of breast cancers [17]. Similarly, in pancreatic adenocarcinoma, the timing of mutations and copy number alterations (CNAs) relative to genome doubling shows that there is a prolonged period of mutational time prior to the duplication event, possibly during states of preinvasive disease, which suggests that subsequent copy number gains accompany transformation [58].

Mutational timing is, therefore, dependent on sufficient numbers of point mutations, which can be problematic, particularly in cancers with low mutation rates. In cases of WGD, however, the total number of point mutations and smaller CNAs across the entire genome provide ample information for calculating time estimates. This is a significant event in the evolution of cancer, as it provides double the raw material for natural selection to shape, allowing cells to achieve aneuploid states that would otherwise not be tolerated. Therefore, the timing of WGD events during tumor evolution is of key importance. Clonal WGD events show variable timing between cancer types; they appear to be late in the evolution of breast cancer [28] and earlier in others, such as colorectal cancer, where it is thought they are the first step in the development of more complex genomic karyotypes, driving disease progression and adversely affecting survival outcomes [59].

Pan-cancer, studies of WGD timing across The Cancer Genome Atlas dataset have demonstrated that the timing of genome doubling relative to both single nucleotide variants (SNVs) and small CNAs is earlier in cancer types with more frequent doubling events, such as ovarian, bladder, and colorectal cancer, compared with those with fewer genome doubling events, such as glioblastoma and clear cell renal carcinoma [60]. This reinforces the idea that, in some cancer types, a tetraploid state is an important milestone for subsequent genomic aberration, whereas in others it is perhaps a product of the accumulation of other CNAs and the loss of DNA maintenance and repair.

In summary, by using the relationships between somatic events it is possible to extract timing of events during tumor evolution from single samples. However, these approaches have only been applied to relatively small cohorts of individual cancer types, and there is still much to be learnt from exploring more general patterns of tumor evolution, pan-cancer.

## Aggregating timing estimates from single samples across cohorts

Cancer evolution is inherently stochastic and, as such, tumor samples within a cohort will have inevitably acquired different sets of mutations, often over different timescales. Thus, the underlying somatic pathways of tumor evolution can be difficult to observe by timing individual events in separate samples. Nevertheless, aggregating temporal relationships between events across a cohort does allow the inference of a common ordering, even where this is not explicitly observed in the data.

This was, in fact, the approach of Fearon and Vogelstein, in their canonical study of the evolution of colorectal cancer [8]. At the most basic level, the temporal ordering of somatic mutations can be inferred from their frequency across a cohort, with samples from various stages of tumor progression; events that are shared by all samples may be considered to have been acquired early, and those which are common only to a subset of more advanced disease stages are assumed to be late events. In more recent years, studies have developed this concept further with the application of graph models in which partial orderings are obtained through the aggregation of genotypes for specific mutations across multiple samples [61–63]. A caveat of these approaches is that the frequency of a mutation cannot always be used as a proxy for the time of its occurrence and, in these cases, the assumptions underlying such models may be considered invalid.

**Fig. 2:**
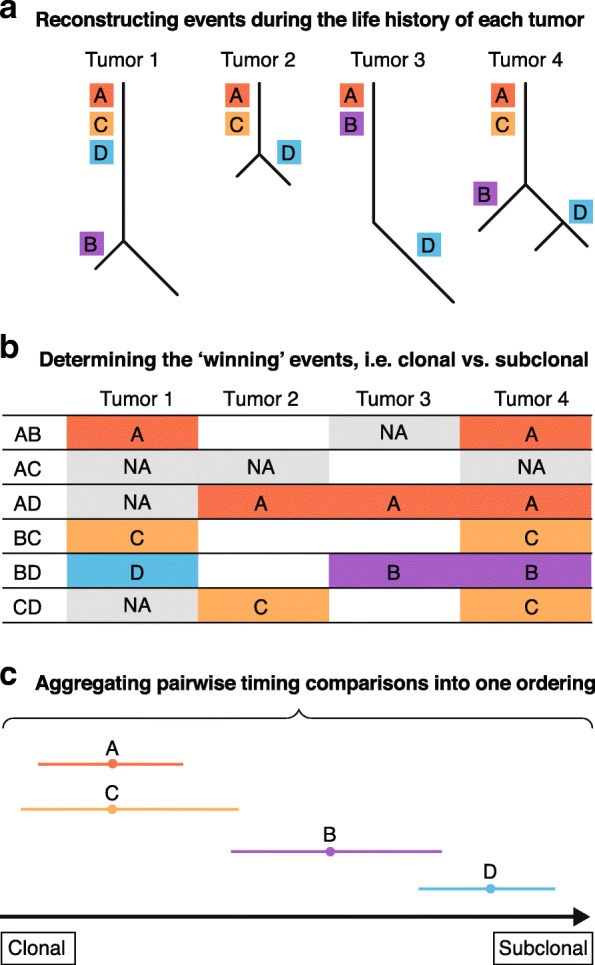
Aggregating the relative timing of events across samples. Once the timing of events within single samples has been established, partial orderings may be aggregated across a cohort to determine an average sequence of events. **a** Example phylogenetic trees which may be reconstructed from tumor life history analyses. Mutations A–D are highlighted on the tree based on their clonal frequency. **b** The outcome of pairwise comparisons between events within each sample, with the “winner” being the earliest event. Comparisons marked “NA” indicate cases where both events are present, but cannot be timed relative to one another. The final ordering: A and C cannot be timed against one another, but win against every other event, indicating that they are the earliest. B has an intermediate ranking, often earlier than D, but never before C or A. D is estimated to be last, as it only once wins a comparison (with B)

More recent studies have made use of the inferred timing of mutations within samples, for example the distinction between clonal and subclonal events, aggregating this information using a sports statistics approach, such as the Bradley–Terry model [64–66]. This type of model is typically applied to the ranking of sports teams within a tournament; teams play against one another, the outcome of which is used to determine an overall ranking from best to worst (see Fig. 2). In the case of cancer genomes, mutations that occur together in one sample can be timed relative to one another, and these pairwise comparisons are aggregated to give an overall ordering of somatic events for a specific cohort or cancer type. In myelodysplastic syndromes, this approach has been used to order mutational events and the underlying pathways; initial mutations are often in genes involved with RNA splicing or DNA methylation, and relatively later or subclonal mutations are more likely to affect chromatin modification or signaling [64]. Applied to prostate cancer [66], early events in ETS^+^ tumors include *TMPRSS2-ERG* fusions and gain of chromosome 8, while losses of chromosomes 5, 13, and 6 are predicted to be the first in the ETS^−^ subtype, followed by losses of chromosome 2 and gains of chromosomes 3 and 7. In both, homozygous deletions are amongst the last events.

This type of timing analysis should give more reliable rankings of somatic mutations during evolution, as the relative timing of clonal and subclonal mutations gives a definite ordering within samples. To date, however, these models have only been applied to a limited number of cancer types and have yet to be validated, for example with time series data.

## Timing the activity of mutational processes

Somatic mutations acquired throughout the course of tumor evolution are the result of a diverse range of mutagenic forces shaping the genome. Next-generation sequencing provides a catalogue of the total somatic mutations acquired by a tumor, which acts as a record of the mutational processes operative throughout its evolutionary past [67]. In a landmark study in 2013, Alexandrov et al. [68] extracted signatures of these mutational processes from a set of five million mutations across 7000 tumors from The Cancer Genome Atlas. Mutations are defined according to their trinucleotide context, generating 96 mutational features to which non-negative matrix factorization was applied. This resulted in the definition of 30 mutational signatures, each one comprising varying proportions of the 96 features. In many instances, these signatures generated mutational profiles reflecting known biological processes. For example, Signature 4 is largely composed of strand-biased C to A transversions, which likely derive from transcription-coupled nucleotide excision repair of bulky DNA adducts caused by tobacco smoking [68–70].

**Fig. 3:**
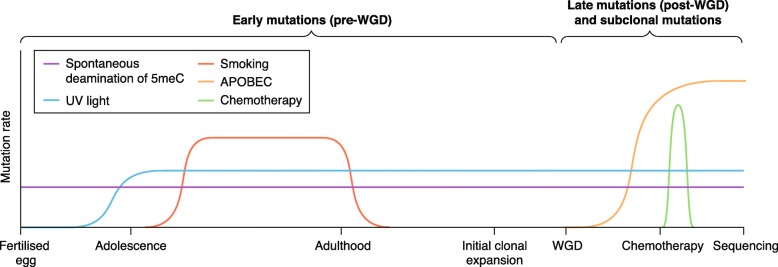
The changing activity of mutational processes during cancer evolution. Mutational forces that operate on the cancer genome are active over different timescales, with varying intensities. A schematic timeline indicates how they correspond to events in a patient’s lifetime. Spontaneous deamination (purple) is operative from the fertilized egg, and is thought to remain relatively constant over time. Exposures to mutagens may also be constant, such as UV light (blue), or transient, such as smoking (red). Tumor-specific processes, such as APOBEC-mediated mutagenesis, become dominant as the tumor develops (yellow) and, finally, the mutational imprint of chemotherapy (green) may be observed in the final few years before sequencing. SNV single nucleotide variant, WGD whole genome duplication

Mutational influences on the genome change throughout tumor evolution (see Fig. 3). Some processes are inherent to all cells and operate constantly, whereas other processes fluctuate as cells are exposed to exogenous mutagens, or as DNA repair processes lose functionality through mutation. The timing of individual point mutations based on clonality and copy number, as described previously, provides one way to study these fluctuations, as the underlying mutational signatures can be extracted from groups of timed mutations [17]. This can be done using one of a number of algorithms developed in recent years for determining the active signatures of mutational processes in separate tumor cohorts; either by re-computing cohort-specific signatures de novo (which may be compared to those established by Alexandrov et al.) [71], or by quantifying the signatures as described in COSMIC [72, 73]. The latter methods have employed both multiple linear regression [74] and probabilistic approaches, based on the expectation-maximization (EM) algorithm [75].

A complementary technique for extracting temporal patterns of mutational signature activity, developed by Rubanova et al. [76], bins mutations using a sliding window approach, first ordering mutations by their cancer cell fraction and then extracting the signatures of mutational processes from sets of 400 mutations. The advantage of this method is that is does not rely on the dependency between point mutations and copy number, or the definitions of clonal and subclonal, to estimate signature changes over time.

In breast cancer, the timing of mutational processes has demonstrated that a signature dominated by C to T mutations at CpG loci, now defined as Signature 1, plays an important role in the acquisition of early mutations and likely corresponds to spontaneous deamination of 5-methyl cytosine, with a more diverse range of mutational spectra taking over in the later and subclonal stages of tumor evolution [17]. In lung cancer, Signature 4 was shown to be active in the early stages of tumor development, but decreased over time, eventually to become superseded by Signatures 2 and 13 (derived from APOBEC-mediated mutagenesis) later in tumor evolution [15, 55]. Thus, it appears that, in the early stages of tumor development, mutations largely derive from intrinsic cellular processes, such as the deamination of methylated cytosine, or from exposure to mutagenic agents such as those found in tobacco smoke. As cancer progresses, the activation of tumor-specific mutational forces, such as the AID/APOBEC cytidine deaminases or defective mismatch repair, contributes proportionally more to the increasing mutational load. This may reflect both the increased deregulation of DNA maintenance and repair processes, but also the growth of the tumor away from the initial source of mutation.

## Deciphering the trajectories of cancer evolution

Computational methods developed in recent years are allowing unprecedented insight into the evolution of cancer from next-generation sequencing data: characterizing clonal dynamics, the timing of mutations, and the changing activity of mutational processes. Various sampling strategies provide complementary approaches for studying the cancer genome, and converge on similar trajectories of evolution. Early events may be defined in various ways: by their presence in precursor lesions, their clonality across different tumor time points or regions, or by their timing relative to other genomic events. Consistently early drivers have been observed in numerous cancer types, and include canonical driver mutations such as *TP53*, *EGFR*, *BRAF*, *PIK3CA*, etc. [15, 17, 40, 55]. There is more diversity in the later stages of evolution, represented by relapse/metastasis-specific events, events occurring after doubling, or subclonal events. These include events related to treatment, such as platinum-resistance mutations in ovarian cancer [34], to the process of metastasis (*JAK2*, *STAT3* in the breast) [28], or more general alterations such as mutations to chromatin remodeling pathways observed in myelodysplasia [64], or the activation of oncogenes in the development of esophageal cancer [40].

Multisample studies provide a direct approach for observing tumor evolution, with higher resolution for reconstructing tumor phylogenies, but exome or targeted sequencing may not always be suitable for analyses requiring large numbers of single nucleotide variants, such as the mutational timing of gains. As was recently discussed [77], the ideal approach may be to take multiple regions of individual tumors, each with deep sequencing data, although currently this is not widely available. Single samples represent a limited region of the tumor; nevertheless, they can be much more easily obtained, and still provide a wealth of information about a tumor’s life history. These analyses are becoming increasingly widespread when studying the tumor genome, and are giving novel insights into the process of tumorigenesis. In the coming years, applying these algorithms to larger datasets should continue to expand our understanding of this process.

To further the clinical relevance of the derived timing of mutations, it would be desirable to know when they occur in real time during a patient’s lifetime. To achieve this, one would need to calibrate time estimates with a molecular clock that is constant throughout normal somatic growth and tumor evolution. One proxy for this is simply the overall mutation burden, which has been shown to correlate with patient age at diagnosis, at least in certain tumor types. This approach allowed the real-time timing of WGD and the emergence of the MRCA in clear cell renal carcinoma, placing these major events many years before diagnosis [78]. Intriguingly, in many tissues, it is the number of mutations generated by mutational Signatures 1 and 5 that appear to correlate best with patient age at diagnosis [79]. The etiology of Signature 1 is considered to be established as spontaneous deamination of methylated cytosines, and is therefore characterized by a high proportion of C to T transitions in a CpG context. However, little is known about Signature 5, which comprises low proportions of most of the 96 mutational features. It appears to be associated with DNA damage from external mutagens, particularly when nucleotide excision repair is deficient, but the underlying mechanism and the interplay between these factors has yet to be elucidated [80]. Thus, Signature 1 may be extracted clearly from other mutation types, and provides a suitable candidate for a real-time mutational clock.

A greater understanding of the temporal sequence of events leading up to tumor formation should allow better prediction of cancer progression, and identification of the earliest, potentially transformative mutations. These events may represent the first steps towards cancer, and so could be used as biomarkers for earlier diagnosis, and possible targets for treatment. In the early stages, however, few cells will bear the genomic lesion, and so it remains a challenge for the future to identify these pre-malignant populations and separate them from normal cells.

## **Box 1:** Deriving time estimates for the acquisition of copy number gains using point mutations

Copy number gains may be timed using point mutations that have accumulated within the gained region [54, 56]. Clonal tumor evolution may be split into time before the chromosomal gain (π_0_) and time after the gain (π_1_), with π_0_ + π_1_ = 1. During π_0_, *x* mutations occur per chromosome copy, while during π_1_, *y* mutations occur per chromosome copy. Therefore, π_0_ may be calculated as the fraction of mutations before the gain, out of the total mutations, i.e., *x*/(*x* + *y)*. In regions of copy-neutral loss of heterozygosity, accounting for the number of chromosomes present during each stage, the observed mutations on two chromosomes (CN2) = *x*, while single copy mutations (CN1) = 2*y*. Put another way, the mutations before the gain (*x*) = CN2, and the mutations after the gain (*y*) = CN1/2. Therefore, π_0_ may be estimated as:

$$ {\pi}_0=\mathrm{CN}2/\left(\mathrm{CN}2+\left(\mathrm{CN}1/2\right)\right) $$

In regions of single gains, where the non-duplicated allele is retained, π_0_ is still calculated in the same way *x*/(*x* + *y*). Now, CN2 still represents *x* (mutations before the gain), but CN1 is the total of post-duplication mutations on the gained allele, pre-duplication mutations on the non-gained allele, and post-duplication mutations on the non-gained allele, i.e., 2*y* + *x* + *y*. Or, *x* = CN2, and *y* = (CN1 – CN2)/3. In this case, π_0_ may be estimated as:

*π*_0_ = CN2/(CN2 + (CN1 − CN2)/3)).

## Abbreviations

CNA: Copy number alteration
SNV: Single nucleotide variant
WGD: Whole genome duplication
